# Supplementary material for: NCKAP1 Inhibits the Progression of Renal Carcinoma via Modulating Immune Responses and the PI3K/AKT/mTOR Signaling Pathway
Source: Int J Mol Sci. 2025 Mar 20;26(6):2813. doi: 10.3390/ijms26062813 (PMC11942877; doi:10.3390/ijms26062813)

## Supplemental Information

# Nck-associated protein NCKAP1 Inhibits the Progression of Renal Carcinoma *via* Modulating Immune Responses and the PI3K/AKT/mTOR Signaling Pathway

Xin Zhang[1], Jianqing Ye[2], Lixiang Sun[1], Wanli Xu[1], Xiaomeng He[3], Juan Bao[3], Jin Wang[1,*]

[1]Central Laboratory, Zhongshan Hospital (Xiamen), Fudan University, Xiamen 361015, Fujan, China; [2]Department of Urology, Xinhua Hospital, School of Medicine, Shanghai Jiaotong University, 1665 Kongjiang Road, Shanghai, China; [3]Shanghai Public Health Clinical Center, Fudan University, 2901 Caolang Road, Jinshan District, Shanghai 201508, China.

***Correspondence should be addressed to:**

Jin Wang, Ph.D.
Central Laboratory,
Zhongshan Hospital (Xiamen),
Fudan University,
Xiamen 361015,
China
Ph: 86-17701869068;
Email: wang.jin@zsxmhospital.com
ORCID ID: orcid.org/0000-0002-0062-2489

## 1. Supplementary Tables

**1.1. Table S1**. The 30 most significantly up-regulated and down-regulated genes in NCKAP1 differential expression analysis.

| Gene id | NCKAP1 | Emp | Fold change | P value | Gene name | Description |
|---|---|---|---|---|---|---|
| ENSG00000283795 | 82.492 | 0.664 | 124.17 | 1.09E-06 | MIR4426 | microRNA 4426 |
| ENSG00000174951 | 1.121 | 0.026 | 43.40 | 2.98E-22 | FUT1 | fucosyltransferase 1 |
| ENSG00000090382 | 2.705 | 0.081 | 33.32 | 0.006718 | LYZ | lysozyme |
| ENSG00000171345 | 1.386 | 0.053 | 26.27 | 0.00017 | KRT19 | keratin 19 |
| ENSG00000211592 | 5.322 | 0.216 | 24.62 | 0.00357 | IGKC | immunoglobulin kappa constant |
| ENSG00000284525 | 12.453 | 0.523 | 23.80 | 0.001598 | MIR4687 | microRNA 4687 |
| ENSG00000124107 | 1.088 | 0.049 | 22.00 | 0.006097 | SLPI | secretory leukocyte peptidase inhibitor |
| ENSG00000019582 | 1.353 | 0.063 | 21.48 | 6.28E-18 | CD74 | CD74 molecule |
| ENSG00000284118 | 10.149 | 0.523 | 19.40 | 0.002757 | MIR4707 | microRNA 4707 |
| ENSG00000189060 | 1.991 | 0.112 | 17.78 | 9.43E-05 | H1-0 | H1.0 linker histone |
| ENSG00000168542 | 2.618 | 0.149 | 17.63 | 0.029343 | COL3A1 | collagen type III alpha 1 chain |
| ENSG00000206337 | 1.905 | 0.112 | 17.06 | 4.31E-15 | HCP5 | HLA complex P5 |
| ENSG00000233929 | 3.619 | 0.225 | 16.09 | 0.002281 | MT1XP1 | metallothionein 1X pseudogene 1 |
| ENSG00000285160 | 1.756 | 0.114 | 15.44 | 0.001274 | THSD1P1 | thrombospondin type 1 domain containing 1 pseudogene 1 |
| ENSG00000108821 | 2.960 | 0.230 | 12.85 | 0.000497 | COL1A1 | collagen type I alpha 1 chain |
| ENSG00000159403 | 3.173 | 0.253 | 12.53 | 2.37E-29 | C1R | complement C1r |
| ENSG00000187953 | 1.981 | 0.163 | 12.17 | 0.002171 | PMS2CL | PMS2 C-terminal like pseudogene |
| ENSG00000289313 | 1.038 | 0.086 | 12.11 | 1.39E-09 | LINC01512 | long intergenic non-protein coding RNA 1512 |
| ENSG00000266933 | 1.209 | 0.103 | 11.77 | 0.000489 | MADCAM1-AS1 | MADCAM1 antisense RNA 1 |
| ENSG00000141756 | 1.801 | 0.159 | 11.34 | 5.58E-20 | FKBP10 | FKBPprolyl isomerase 10 |
| ENSG00000165949 | 65.480 | 5.835 | 11.22 | 2.21E-58 | IFI27 | interferon alpha inducible protein 27 |
| ENSG00000249992 | 7.131 | 0.637 | 11.19 | 1.12E-18 | TMEM158 | transmembrane protein 158 |
| ENSG00000226972 | 1.914 | 0.177 | 10.83 | 0.012053 | RPL12P19 | ribosomal protein L12 pseudogene 19 |
| ENSG00000141753 | 2.374 | 0.219 | 10.83 | 2.79E-05 | IGFBP4 | insulin like growth factor binding protein 4 |
| ENSG00000230191 | 1.093 | 0.102 | 10.74 | 0.010918 | IFITM3P4 | IFITM3 pseudogene 4 |
| ENSG00000239528 | 11.829 | 1.116 | 10.60 | 0.000669 | RPS14P8 | ribosomal protein S14 pseudogene 8 |
| ENSG00000095303 | 2.007 | 0.191 | 10.52 | 2.51E-17 | PTGS1 | prostaglandin-endoperoxide synthase 1 |
| ENSG00000278763 | 1.189 | 0.117 | 10.15 | 0.01794 | FAM27B | family with sequence similarity 27 member B |
| ENSG00000198625 | 1.809 | 0.184 | 9.86 | 2.73E-07 | MDM4 | MDM4 regulator of p53 |
| ENSG00000149150 | 1.714 | 0.176 | 9.76 | 2.56E-14 | SLC43A1 | solute carrier family 43 member 1 |
| ENSG00000072274 | 4.295 | 11.824 | -2.75 | 2.79E-15 | TFRC | transferrin receptor |
| ENSG00000115474 | 0.834 | 2.304 | -2.76 | 1.76E-08 | KCNJ13 | potassium inwardly rectifying channel subfamily J member 13 |
| ENSG00000112984 | 4.123 | 11.405 | -2.77 | 3.53E-15 | KIF20A | kinesin family member 20A |
| ENSG00000228716 | 2.622 | 7.285 | -2.78 | 7.33E-13 | DHFR | dihydrofolate reductase |
| ENSG00000024526 | 2.173 | 6.070 | -2.79 | 2.00E-13 | DEPDC1 | DEP domain containing 1 |
| ENSG00000271092 | 1.178 | 3.331 | -2.83 | 5.42E-06 | TLCD4-RWDD3 | TLCD4-RWDD3 readthrough |
| ENSG00000263961 | 0.370 | 1.048 | -2.83 | 0.00032 | RHEX | regulator of hemoglobinization and erythroid cell expansion |

| | | | | | | |
|---|---|---|---|---|---|---|
| ENSG00000139163 | 0.761 | 2.161 | -2.84 | 0.001955 | ETNK1 | ethanolamine kinase 1 |
| ENSG00000080824 | 71.339 | 202.646 | -2.84 | 1.24E-17 | HSP90AA1 | heat shock protein 90 alpha family class A member 1 |
| ENSG00000029363 | 1.320 | 3.798 | -2.88 | 4.17E-17 | BCLAF1 | BCL2 associated transcription factor 1 |
| ENSG00000196227 | 0.710 | 2.050 | -2.89 | 2.89E-06 | FAM217B | family with sequence similarity 217 member B |
| ENSG00000112245 | 3.005 | 8.849 | -2.94 | 0.001611 | PTP4A1 | protein tyrosine phosphatase 4A1 |
| ENSG00000228253 | 4181.160 | 12325.896 | -2.95 | 4.19E-21 | MT-ATP8 | mitochondrially encoded ATP synthase membrane subunit 8 |
| ENSG00000115221 | 0.958 | 2.838 | -2.96 | 1.42E-10 | ITGB6 | integrin subunit beta 6 |
| ENSG00000167460 | 5.174 | 15.459 | -2.99 | 4.79E-11 | TPM4 | tropomyosin 4 |
| ENSG00000224578 | 21.195 | 63.485 | -3.00 | 4.05E-06 | HNRNPA1L3 | heterogeneous nuclear ribonucleoprotein A1 like 3 |
| ENSG00000204389 | 3.547 | 10.705 | -3.02 | 0.003119 | HSPA1A | heat shock protein family A (Hsp70) member 1A |
| ENSG00000260804 | 2.009 | 6.069 | -3.02 | 2.01E-10 | LINC01963 | long intergenic non-protein coding RNA 1963 |
| ENSG00000242265 | 1.890 | 5.711 | -3.02 | 3.98E-08 | PEG10 | paternally expressed 10 |
| ENSG00000198585 | 0.518 | 1.592 | -3.08 | 0.000811 | NUDT16 | nudix hydrolase 16 |
| ENSG00000248527 | 347.811 | 1091.468 | -3.14 | 1.13E-05 | MTATP6P1 | MT-ATP6 pseudogene 1 |
| ENSG00000183444 | 1.301 | 4.089 | -3.14 | 0.008222 | OR7E38P | olfactory receptor family 7 subfamily E member 38 pseudogene |
| ENSG00000168497 | 21.441 | 68.368 | -3.19 | 3.70E-20 | CAVIN2 | caveolae associated protein 2 |
| ENSG00000119616 | 3.200 | 11.002 | -3.44 | 6.47E-19 | FCF1 | FCF1 rRNA-processing protein |
| ENSG00000185414 | 1.538 | 5.336 | -3.47 | 3.21E-09 | MRPL30 | mitochondrial ribosomal protein L30 |
| ENSG00000284981 | 0.356 | 1.336 | -3.75 | 0.012478 | UPK3BL2 | uroplakin 3B like 2 |
| ENSG00000276788 | 1.241 | 5.109 | -4.12 | 0.012757 | SNORD26 | small nucleolarRNA, C/D box 26 |
| ENSG00000200879 | 0.217 | 1.820 | -8.40 | 0.048164 | SNORD14E | small nucleolarRNA, C/D box 14E |
| ENSG00000108825 | 0.052 | 1.813 | -35.04 | 0.002936 | PTGES3L-AARSD1 | PTGES3L-AARSD1 readthrough |
| ENSG00000243509 | 0.049 | 16.378 | -332.38 | 1.43E-06 | TNFRSF6B | TNF receptor superfamily member 6b |

**1.2. Table S2**. Key pathways and their corresponding genes identified among the top 30 differential expressed genes in KEGG pathway enrichment analysis.

| Pathway | HIF-1 signaling pathway | AMPK signaling pathway | MAPK signalig pathway | p53 signaling pathway | TNF signaling pathway | PI3K-Akt signaling pathway | JAK-STAT signaling pathway |
|---|---|---|---|---|---|---|---|
| UP genes | EDN1 | | | MDM4 | CCL5<br>EDN1 | COL1A1 | |
| Down genes | TFRC | LEPR | HSPA1A<br>HSPA8 | CCNE2 | | ITGB6<br>CCNE2<br>HSP90AA1 | LEPR |

## 2. Supplementary Figures

2.1 **Supplemental Figure S1**. Expression of NCKAP1 in KIRC, KIRP, and KICH and Its Association with Prognosis. (A) Expression of NCKAP1 in paired and unpaired samples of KIRC, KIRP, and KICH. (B) The association of NCKAP1 expression with overall survival (OS), disease-specific survival (DSS), and progression-free interval (PFI) in KIRC, KIRP, and KICH.

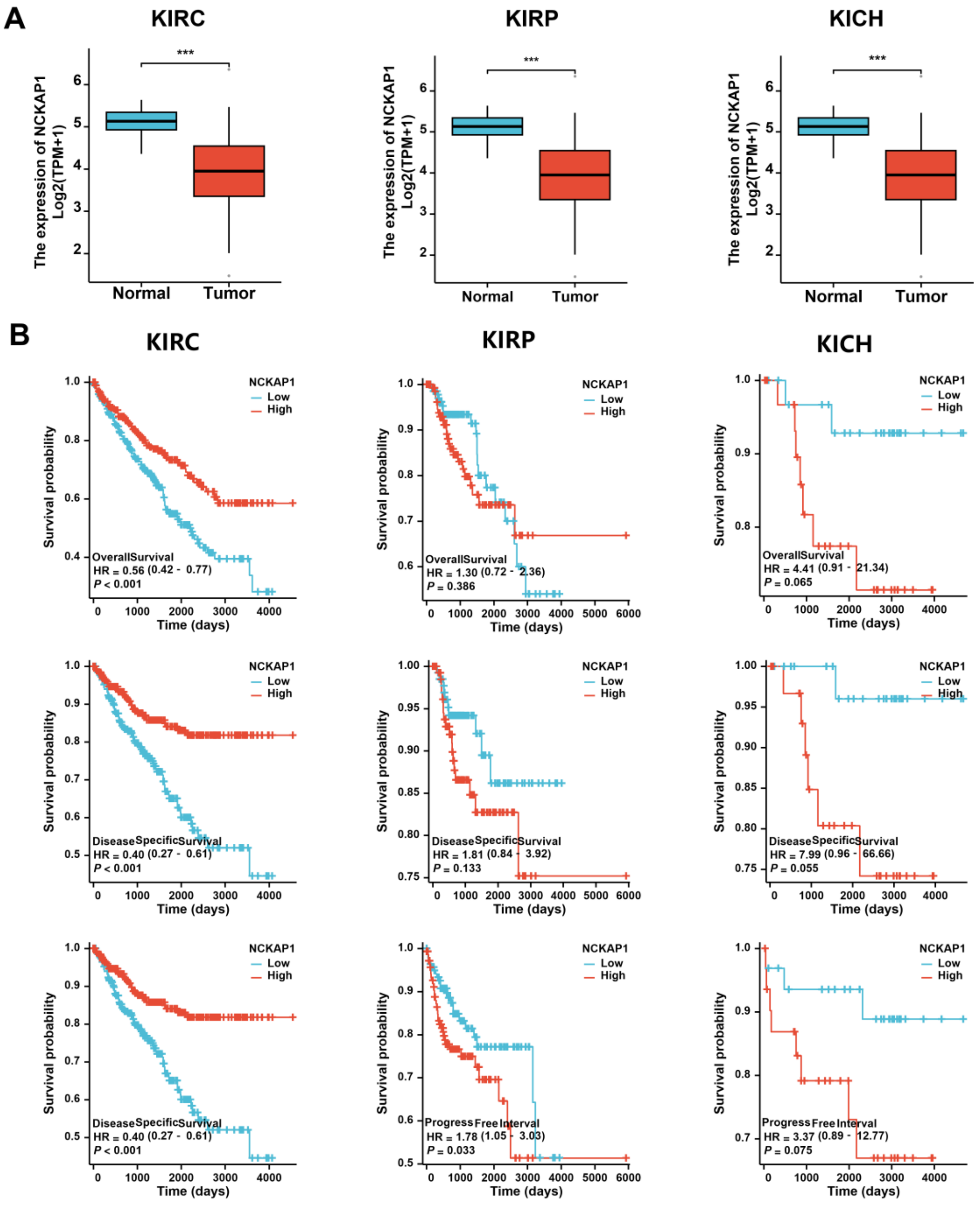

**2.2 Supplemental Figure S2**. A PPI network comprising 50 experimentally validated NCKAP1-interacting genes was visualized using the STRING web server platform.

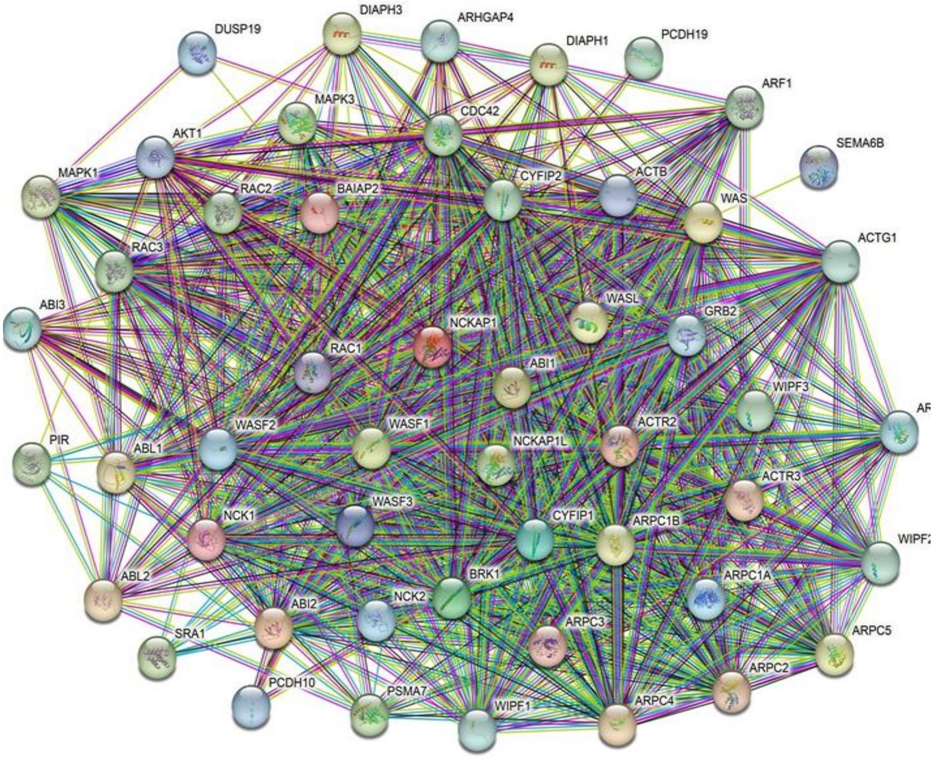

**2.3. Supplemental Figure S3. Functional Enrichment Analysis of NCKAP1- Related and Differentially Expressed Genes.** (A) The heatmap illustrates the expression levels of genes such as CAMSAP1, RAB3GAP1, SCRN3, SP3, SPTLC1 and ZFP91 across various cancer types. (B) Venn diagram illustrating the intersection analysis of genes that interact with NCKAP1 and those that correlate with NCKAP1 expression. (C) Bubble chart demonstrating the results of GO and KEGG analyses for differential NCKAP1 expression in renal cancer. (D) GSEA of NCKAP1 expression in KICH. (E) GSEA of NCKAP1 in KIRC.

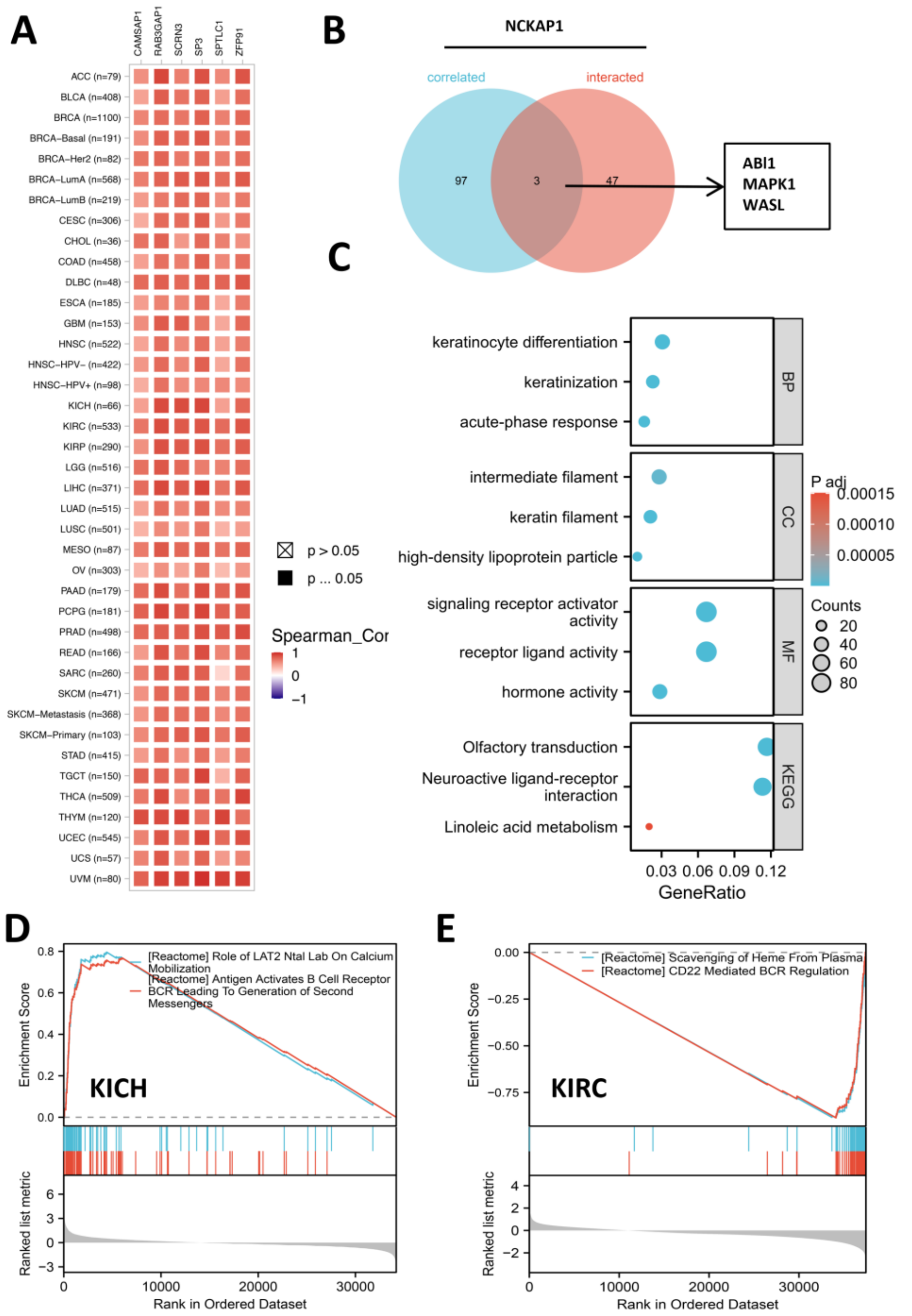

Supplement: Supplementary file 1 [file ijms-26-02813-s001.zip › ijms-3467526-supplementary.pdf]
